# A *Bursaphelenchus xylophilus* Effector, BxSCD3, Suppresses Plant Defense and Contributes to Virulence

**DOI:** 10.3390/ijms23126417

**Published:** 2022-06-08

**Authors:** Long-Jiao Hu, Xiao-Qin Wu, Tong-Yue Wen, Yi-Jun Qiu, Lin Rui, Yan Zhang, Jian-Ren Ye

**Affiliations:** 1 Co-Innovation Center for Sustainable Forestry in Southern China, College of Forestry, Nanjing Forestry University, Nanjing 210037, China; longjiaohu812@163.com (L.-J.H.); tywen940822@126.com (T.-Y.W.); qiuyijun722@126.com (Y.-J.Q.); ruilin0305@njfu.edu.cn (L.R.); yanzhang5257@163.com (Y.Z.); jrye@njfu.edu.cn (J.-R.Y.); 2 Institute of Botany, Jiangsu Province and Chinese Academy of Sciences, Nanjing 210014, China

**Keywords:** *Bursaphelenchus xylophilus*, effector, suppresses plant defense, defense-related genes, *Pinus thunbergii*

## Abstract

*Bursaphelenchus xylophilus* is the most economically important species of migratory plant-parasitic nematodes (PPNs) and causes severe damage to forestry in China. The successful infection of *B. xylophilus* relies on the secretion of a repertoire of effector proteins. The effectors, which suppress the host pine immune response, are key to the facilitation of *B. xylophilus* parasitism. An exhaustive list of candidate effectors of *B. xylophilus* was predicted, but not all have been identified and characterized. Here, an effector, named BxSCD3, has been implicated in the suppression of host immunity. BxSCD3 could suppress pathogen-associated molecular patterns (PAMPs) PsXEG1- and INF1-triggered cell death when it was secreted into the intracellular space in *Nicotiana benthamiana. BxSCD3* was highly up-regulated in the early infection stages of *B. xylophilus*. *BxSCD3* does not affect *B. xylophilus* reproduction, either at the mycophagous stage or the phytophagous stage, but it contributes to the virulence of *B. xylophilus.* Moreover, *BxSCD3* significantly influenced the relative expression levels of defense-related (PR) genes *PtPR-3* and *PtPR-6* in *Pinus thunbergii* in the early infection stage. These results suggest that BxSCD3 is an important toxic factor and plays a key role in the interaction between *B. xylophilus* and host pine.

## 1. Introduction

An important migratory plant-parasitic nematode (PPN) *Bursaphelenchus xylophilus* causes pine wilt disease (PWD). *B. xylophilus* is native to North America and it causes little damage to native trees in America [1]. However, it was introduced into East Asia (including China, Japan and Korea) at the start of the 20th century, which has resulted in increasingly serious economic losses and ecological damage under appropriate environmental conditions, especially in China and Japan [2,3]. The biology and the life cycle of *B. xylophilus* have been reviewed and summarized in detail [4,5]. *B. xylophilus* has two different life cycle stages—the phytophagous stage and the mycetophagous stage. When *B. xylophilus* juveniles are spread to healthy pine trees, they feed on nutrients from pine tissues. However, when the tree wilts or dies, they can feed on abundant fungi in the tree. This unique feature distinguishes it from other PPNs.

Like other pathogenic microbes, to achieve successful host colonization, *B. xylophilus* must overcome plant immunity [6]. Generally, the plant’s innate immune system has two layers. Firstly, pathogen- or microbe-associated molecular patterns (PAMPs or MAMPs, respectively) are recognized by plant plasma membrane-bound receptors (pattern recognition receptors (PRRs)) to induce the first tier of innate immunity (PAMP-triggered immunity (PTI)) [7]. In turn, the pathogens secrete effectors to suppress PTI, facilitating infection. The plants employ the nucleotide-binding and leucine-rich repeat (NB-LRR) proteins encoded by disease resistance (R) genes to recognize effectors and trigger the second overlapping mode of innate immunity (effector-triggered immunity (ETI)) [6,8]. Many studies have shown that the effectors of various pathogens contribute to virulence in the interaction of pathogens and plants, such as oomycetes, fungi, bacteria, and PPNs [9,10,11,12].

The phytonematodes include sedentary and migratory PPNs, and whatever the kind of nematode, they all need to feed on viable host cells for nutrition via the stylet [13]. Thus, efficient mechanisms must be employed to suppress or evade host defenses at this stage. There is increasing evidence that PPNs harbor a significant number of effectors that are involved in protection against host defenses [11,14]. Many phytonematodes deliver effectors into host cells to suppress immune responses as a form of parasitism, including PAMP-triggered or proapoptotic mouse protein BAX-triggered programmed cell death(PCD) [15,16]. The ability to suppress PAMP-triggered or BAX-triggered PCD has proven to be a valuable initial screening method for microbial plant pathogen effectors. For example, the *Phytophthora sojae* effector Avh238 could suppress INF1-triggered cell death in *Nicotiana benthamiana* [7]. A cyst nematode *Heterodera avenae* effector Ha-annexin and seven *Valsa mali* effector proteins (VmEPs) could all suppress BAX-triggered cell death, respectively [17,18]. Although efforts have been made to provide an exhaustive list of effectors of *B. xylophilus* [19,20,21], only two effectors, BxSCD1 and Bx-FAR-1, were validated to suppress immune responses [22,23]. Effectors are a class of molecules that act in teams, so it is likely that many other important effectors need to be discovered and characterized.

In this study, BxSCD3 has been implicated in the suppression of host immunity. BxSCD3 could suppress *P. sojea* and *P. infestans* PAMPs PsXEG1- and INF1-triggered cell death when it secreted into the intracellular space in *N. benthamiana.* BxSCD3 plays an inhibitory role in plant immunity whether it is located in the cytoplasm or the nucleus. *BxSCD3* significantly influenced the relative expression levels of defense-related (PR) genes *PtPR-3* and *PtPR-6* in *Pinus thunbergii* in the early infection stage. Moreover, BxSCD3 contributes to the virulence, and does not affect the reproduction of *B. xylophilus* in the process of interaction between *B. xylophilus* and pines.

## 2. Results

### 2.1. BxSCD3 Suppresses PsXEG1- and INF1-Induced Cell Death in N. benthamiana When Secreted into the Intracellular Space

The gene (BXY_0601800) was identified from the transcriptome of *B. xylophilus*, which was up-regulated in the phytophagous phase (2.5 h after inoculation) of *B. xylophilus* [21]. It encodes a 165-amino acid polypeptide with a 15-amino acid signal peptide (SP) at the N terminus, but without a putative transmembrane. It was predicted to contain no known domain using the Simple Modular Architecture Research Tool (SMART).

**Figure 1:**
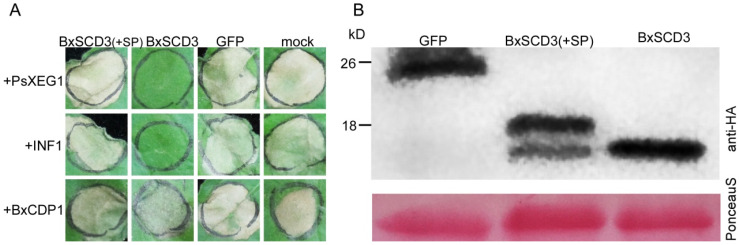
BxSCD3 suppresses PsXEG1- and INF1-induced cell death in *Nicotiana benthamiana* when secreted into the intracellular space. (**A**) Representative *N. benthamiana* leaves at 7 days (d) after inoculation with *Agrobacterium* sp. strain GV3101 carrying BxSCD3 in vector PVX (pGR107). The infiltration assay was performed three times and, in each assay, three different plants with three inoculated leaves were used. (**B**) Immunoblot analysis of proteins from *N. benthamiana* leaves transiently expressing target proteins fused with anti-hemagglutinin (anti-HA) tags.

In a previous study, investigating whether overexpression of pathogen effectors can inhibit PAMP-triggered cell death in *N. benthamiana* is considered a sound strategy for detecting immunosuppressive abilities [24]. PsXEG1, INF1, and BxCDP1 are all PAMPs, which are from *P. sojea*, *P. infestans* and *B. xylophilus*, respectively. Additionally, they can trigger immunity-related hypersensitive responses in various plants including *N. benthamiana* [2,25,26]. To determine the immunosuppressive abilities, the *Agrobacterium* strains carrying the PsXEG1 or INF1 or BxCDP1 construct were infiltrated into *N. benthamiana* leaves, in which BXY_0601800 (with SP), BXY_0601800 (without SP), or green fluorescent protein (GFP) had been expressed 16 h before. The result showed that transiently expressed BXY_0601800 (with or without SP) could not suppress BxCDP1-triggered cell death (Figure 1A). However, BXY_0601800 (without SP) could suppress both PsXEG1- and INF1-triggered cell death (Figure 1A). In addition, the negative control expressing GFP and BXY_0601800 (with SP) did not possess this ability (Figure 1A). The expression of these proteins was validated by Western blot analysis (Figure 1B). The data indicated that BXY_0601800 (without SP) suppresses PAMP PsXEG1- and INF1-triggered cell death in *N. benthamiana* when secreted into the intracellular space. Thus, the protein was chosen for further study and denoted as BxSCD3 (suppresses cell death).

### 2.2. BxSCD3 Plays an Inhibitory Role in Plant Immunity Whether It Is Located in the Cytoplasm or the Nucleus

**Figure 2:**
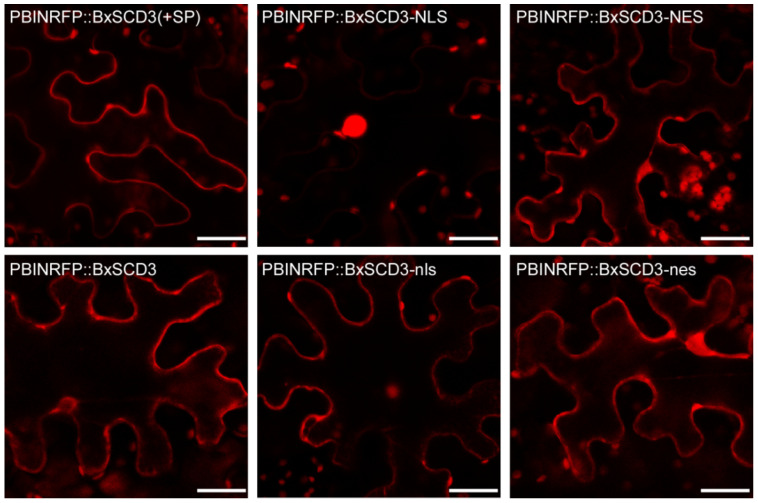
The subcellular localization of BxSCD3 in *Nicotiana benthamiana.* The subcellular localization of BxSCD3, BxSCD3-NLS (nuclear localization signal), BxSCD3-NES (nuclear export signal), and two mutant forms, BxSCD3-nls and BxSCD3-nes, were determined by transient expression of red fluorescent protein (RFP)-tagged proteins in *N. benthamiana* leaves. Confocal microscopy images were taken 36 h post-infiltration. Bars = 20 µm.

To determine the subcellular localization of BxSCD3, we expressed N-terminal red fluorescent protein (RFP) pBINRFP-tagged BxSCD3 (without SP) and various mutants in *N. benthamiana*, which included pBINRFP-tagged BxSCD3, directed to the nucleus or the cytoplasm by fusing either a nuclear localization signal (NLS) or a nuclear export signal (NES) to it (BxSCD3-NLS and BxSCD3-NES, respectively), and mutated NLS (BxSCD3-nls) and NES (BxSCD3-nes). The BxSCD3-nls and BxSCD3-nes were used as negative controls. Upon the expression of BxSCD3-RFP in *N. benthamiana* leaves, RFP-derived fluorescence was detected both in the nucleus and the cytoplasm (Figure 2). In addition, BxSCD3-NLS showed exclusive fluorescence in the nucleus, in contrast to BxSCD3-nls, BxSCD3-NES, and BxSCD3-nes, which also showed fluorescence in the cytoplasm (Figure 2). Moreover, BxSCD3-NES showed a strong reduction of fluorescence in the nucleus, indicating that the NES fused to BxSCD3 was, to a certain extent, capable of retaining BxSCD3 in the cytoplasm (Figure 2). The result showed that BxSCD3 is established in the nucleus and also in the cytoplasm.

**Figure 3:**
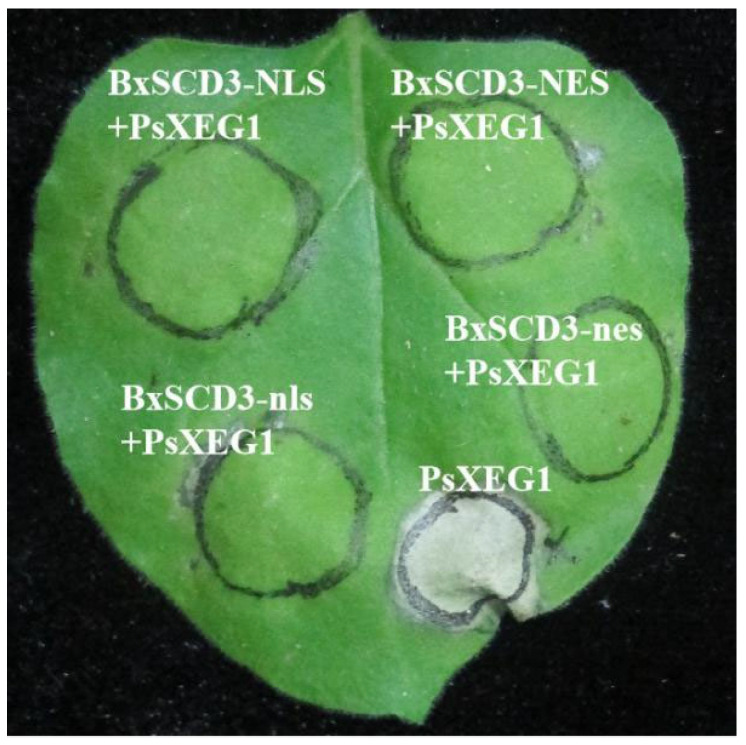
Whether BxSCD3 locates in the cytoplasm or the nucleus, it can suppress PsXEG1-induced cell death in *Nicotiana benthamiana* leaves. BxSCD3-NLS (nuclear localization signal), BxSCD3-NES (nuclear export signal), and two mutant forms, BxSCD3-nls and BxSCD3-nes, were transiently expressed in *N. benthamiana* leaves. The infiltration assay was performed three times and, in each assay, three different plants with three inoculated leaves were used.

Moreover, to investigate which subcellular localization of BxSCD3 is required for the immunosuppressive activity, we also expressed N-terminal PVX-tagged BxSCD3-NLS, BxSCD3-nls, and BxSCD3-nes, respectively. When expressing these BxSCD3 mutants in *N. benthamiana* leaves, we found that these BxSCD3 mutants were all able to suppress cell death, as was the case for BxSCD3 (Figure 3). Cell death induction activity was also monitored using ion leakage, which showed consistent results (Figure 3). Together, these results strongly implied that the nuclear or cytoplasm pools of BxSCD3 were both essential for cell death-inducing activity.

### 2.3. The Expression of BxSCD3 Increased Significantly at the Early Infection Stage

**Figure 4:**
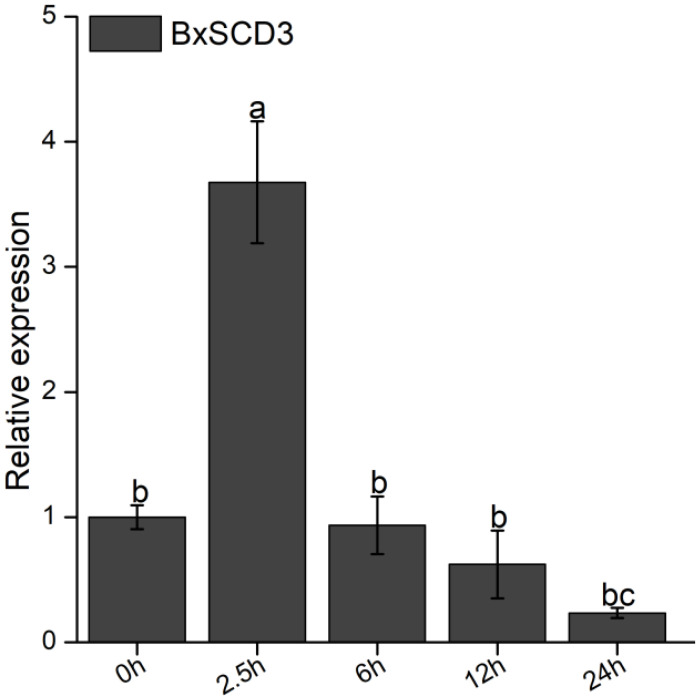
Relative transcript level of *BxSCD3* in *Bursaphelenchus xylophilus* during the early stages of infection. Data are the means, and the error bars represent ± standard deviation from three biological replicates. Different letters on top of the bars indicate statistically significant differences (*p* < 0.05, *t*-test) as measured by Duncan’s multiple range test.

*BxSCD3* was proven to be up-regulated in the transcriptome of *B. xylophilus* (2.5 h post-infection) [21]. To verify the result, we detected the relative expression of *BxSCD3* from the cDNA of *B. xylophilus* by quantitative real-time polymerase chain reaction (RT-qPCR). This showed that *BxSCD3* did indeed increase significantly in *B. xylophilus* at 2.5 h after inoculation (phytophagous stage), compared to the mycophagous stages (Figure 4).

### 2.4. BxSCD3 Does Not Affect B. xylophilus Reproduction, Either at the Mycophagous Stage or the Phytophagous Stage

**Figure 5:**
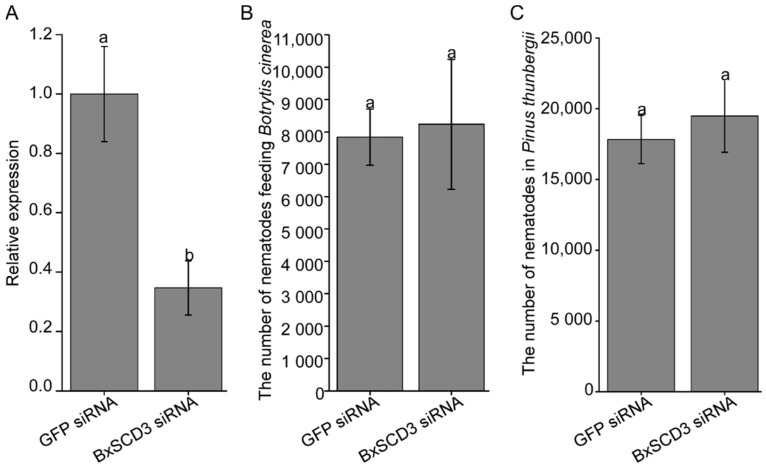
*BxSCD3* does not affect *Bursaphelenchus xylophilus* reproduction. (**A**) The silencing efficiency of *BxSCD3* was measured by quantitative real-time polymerase chain reaction (RT-qPCR). (**B**) The number of *BxSCD3* siRNA-treated nematodes after inoculation into *Botrytis* *cinerea* for 5 days (d). (**C**) The number of *BxSCD3* siRNA-treated nematodes after inoculation into *Pinus* *thunbergii* for 25 d. The *GFP* siRNA-treated nematodes were used as a control in the above three independent experiments. Data are the means, and the error bars represent ± SD from three biological replicates. Different letters on top of the bars indicate statistically significant differences (*p* < 0.05, *t*-test) as measured by Duncan’s multiple range test.

RNA interference (RNAi) was used to explore the contribution of *BxSCD3* to the virulence of *B. xylophilus*. The RT-qPCR analysis showed that the expression of *BxSCD3* in the *BxSCD3* siRNA-treated nematodes was much lower than in the GFP siRNA solutions-treated nematodes, and it verified that *BxSCD3* was silenced successfully (Figure 5A). To investigate the influence of *BxSCD3* on the reproduction of *B. xylophilus*, the numbers of *B. xylophilus* were counted to measure their reproduction after silencing *BxSCD3*. The similar results obtained from the three treatments indicated that *BxSCD3* had little influence on the reproduction of *B. xylophilus* at the mycophagous stages (Figure 5B).

Moreover, the numbers of *B. xylophilus* in the host inoculated with *BxSCD3* siRNA-treated nematodes and GFP siRNA-treated nematodes were also counted when the control seedlings had withered entirely. This showed that the numbers of *B. xylophilus* in seedlings inoculated with *BxSCD3* siRNA-treated nematodes were a little higher than in seedlings inoculated with *GFP* siRNA-treated nematodes, but the difference was not significant (Figure 5C). Thus, these results indicated that *BxSCD3* does not affect *B. xylophilus* reproduction, either at the mycophagous stage or the phytophagous stage.

### 2.5. BxSCD3 Contributes to Virulence during Infection

**Figure 6:**
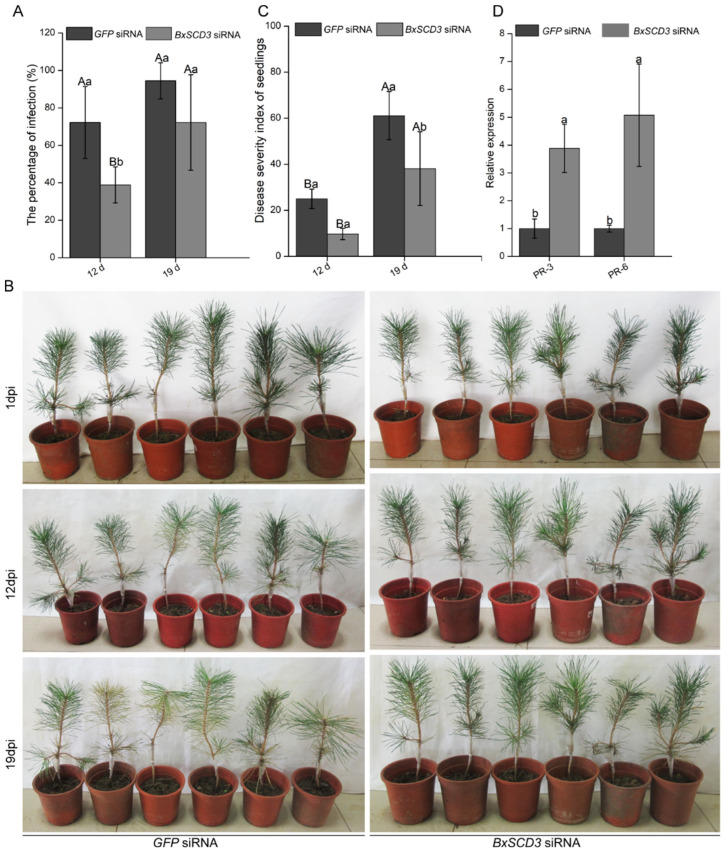
*BxSCD3* contributes to the virulence of *Bursaphelenchus xylophilus* and inhibits the expression of defense-related genes in *Pinus thunbergii*. (**A**) The infection ratio of pine seedlings were calculated at 12 and 19 days post-inoculation (dpi). Three independent experiments were performed, and 18 individual *P. thunbergii* seedlings were used for each treatment. Data are the means, and the error bars represent ± SD from three biological replicates. Different letters on top of the bars indicate statistically significant differences (*p* < 0.05, *t*-test) as measured by Duncan’s multiple range test. (**B**) The symptoms of *P. thunbergii* at 12 and 19 dpi with two different nematode treatments (*BxSCD3* siRNA and *GFP* siRNA). (**C**) The disease severity index of pine seedlings were calculated at 12 and 19 days dpi. Data are the means, and the error bars represent ± SD from three biological replicates. Different letters on top of the bars indicate statistically significant differences (*p* < 0.05, *t*-test) as measured by Duncan’s multiple range test. (**D**) The relative expression levels of pathogenesis-related genes *PtPR-3* and *PtPR-6* in *P. thunbergii* infected with *BxSCD3* siRNA-treated nematodes. The seedlings infected with *GFP* siRNA-treated nematodes were used as controls. Data are the means, and the error bars represent ± SD from three biological replicates. Different letters on top of the bars indicate statistically significant differences (*p* < 0.05, *t*-test) as measured by Duncan’s multiple range test.

The inoculation assay showed that this early symptom occurred later in seedlings inoculated with *BxSCD3* siRNA-treated nematodes (Figure 6B; Supplementary Figures S1 and S2). Moreover, at 12 days (d) and 19 d post-inoculation, the infection ratio and DSI of *P. thunbergii* seedlings inoculated with *BxSCD3* siRNA-treated nematodes were significantly lower than those of seedlings inoculated with *GFP* siRNA-treated nematodes (Figure 6A,C). These results suggested that *B. xylophilus* pathogenicity was significantly reduced when *BxSCD3* was silenced, indicating that *BxSCD3* contributes to the virulence of *B. xylophilus* at the early stages of infection.

### 2.6. BxSCD3 Significantly Influenced the Relative Expression Levels of Defense-Related Genes in P. thunbergii at the Early Infection Stage

We tested whether *BxSCD3* silencing in *B. xylophilus* affects the expression of PR genes in *P. thunbergii*. The RT-qPCR analysis showed that, when *BxSCD3* was silenced, the relative expression levels of *PtPR-3* and *PtPR-6* were significantly higher than in *P. thunbergii* inoculated with *GFP* siRNA-treated nematodes (Figure 6D). This indicated that *BxSCD3* indeed influenced defense responses of *P. thunbergii*, and that it might promote the infection of *B. xylophilus* by inhibiting the expression of *PtPR-3* and *PtPR-6*. 

**Figure 7:**
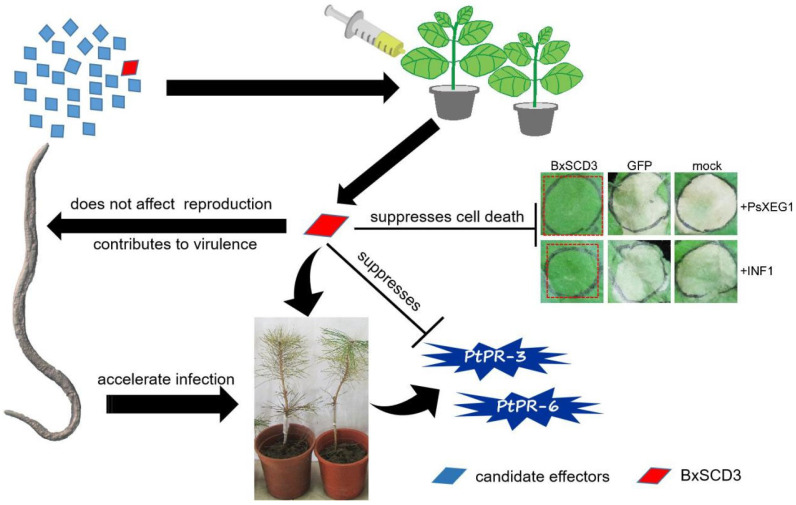
Functional diagram of BxSCD3. BxSCD3 was identified as an effector by agrobacterium-mediated transient expression in *Nicotiana benthamiana*, which suppressed PAMPs PsXEG1- and INF1-triggered cell death. BxSCD3 contributes to the virulence, does not affect reproduction of *Bursaphelenchus xylophilus* in the process of interaction between *B. xylophilus* and pines, and significantly suppresses the relative expression levels of defense-related (PR) genes *PtPR-3* and *PtPR-6* in *Pinus thunbergii*, which accelerates the infection progress of *B. xylophilus*.

Finally, we drew a functional diagram of BxSCD3 to summarize the results of this study (Figure 7).

## 3. Discussion

Many studies have demonstrated the cooperation of effectors in teams, and how effectors with different functions are secreted in the different infection stages to finally promote successful infection [27,28]. In the process of interaction between pathogens and hosts, pathogens secreted PAMPs and effectors to trigger PTI and ETI. At the same time, pathogens also secreted some effectors to inhibit the PTI and ETI and help the pathogens escape host recognition under the strong pressure of natural selection [6]. For example, the famous oomycete *P. sojae* was predicted to have more than 300 effectors carrying RxLR dEER motifs [29], and 169 RXLR effectors from the *P. sojae* in *N. benthamiana* were screened [30]. Among them, many effectors suppressed PCD and/or PAMP INF1- or PsXEG1-triggered cell death and PTI [25,30]. In our previous research, BxCDP1 as a PAMP could induce cell death and PTI of pines [2]. The effector Bx-FAR-1 could suppress BAX- and INF1-triggered cell death [22], and BxSCD1 could suppress BxCDP1-triggered cell death and PTI [23]. In this study, BxSCD3 was identified to suppress cell death induced by PAMP PsXEG1 and INF1, but not by BxCDP1. Meanwhile, the expression of BxSCD3 reached its peak at 2.5 h after inoculation (a very early period of infection), and Bx-FAR-1 and BxSCD3 reached their peaks at 24 h and 12 h after inoculation, respectively. These results indicated that *B. xylophilus*, like other pathogens, secreted multiple effectors that inhibit plant immunity at different infection stages. However, it remains to be found whether PAMPs- or effector-triggered immunity can be inhibited by BxSCD3 in *B. xylophilus*.

Many effectors played a role in intracellular sites that function only in the nucleus or only in the cytoplasm to control plant immunity. For example, the verticillium-specific protein VdSCP7 localizes to the nucleus of plant cells and induces immunity to fungal infection [31]. Cytoplasmic localization of *B. xylophilus* effector BxSCD1 was required for its suppression of cell death [23]. In this study, BxSCD3-pBINRFP accumulated both in nucleus and cytoplasmic locations, and whether BxSCD3 locates in the cytoplasm or the nucleus, it plays an inhibitory role in plant immunity. This result indicated that BxSCD3 is a translocated effector. A similar result was found in *Bipolaris sorokiniana* effector CsSp1, which triggered plant immunity in both the nucleus and the cytoplasm of *N. benthamiana* cells [32]. There was no NLS or other organelle localization signals in CsSp1, so the localization of CsSp1 was predicted to be influenced by the target protein, and CsSp1 target proteins are present in both the nucleus and the cytoplasm of plants [32]. Thus, the localization of BxSCD3 may also be influenced by the target protein, which may be also present in both the nucleus and the cytoplasm of plants. We have tried to screen the target proteins of BxSCD3 using a yeast two-hybrid system, but it unfortunately failed. In a future study, we want to try to explore the accurate location of BxSCD3 in the host, and the target proteins of BxSCD3 will be screened and identified again to further reveal the function mechanism of BxSCD3 in the interaction between *B. xylophilus* and pines. 

Nematodes feed on host cells for nutrition via the stylet, thus, efficient mechanisms must be employed to suppress host defenses at this stage [33]. To achieve this, *B. xylophilus* needs to deliver several effectors into host cells to suppress immune responses, including PTI and the expression of PR genes. In this study, we showed that transient expression of BxSCD3 could suppress PTI triggered by PAMPs PsXEG1 or INF1. At the same time, silencing of BxSCD3 in vitro significantly increased the expressions of *PR* genes *PtPR-3* and *PtPR-6.* Among them, *PtPR-6* is a jasmonic acid (JA) and ethylene-responsive gene [34]. These results indicated that BxSCD3 is important for *B. xylophilus* parasitism, and BxSCD3 expression might interfere with host signaling pathways and immune responses. We will further measure the contents of JA and ethylene in *P. thunbergii* inoculated with *BxSCD3* siRNA-treated nematodes to verify this conjecture in the following study.

In previous studies, several pathogenesis-related genes affected the reproductive ability of *B. xylophilus* to delay the onset of PWD, such as *BxATG1*, *BxATG8*, three *Bx-cpls* and effector Bx-FAR-1 [22,35,36]. In this study, when *BxSCD3* was silenced, the onset of host pine was delayed, but it did not affect the reproduction of *B. xylophilus* either in the phytophagous stage or the mycetophagous stage. This suggested that BxSCD3 was a toxic factor of *B. xylophilus*, rather than helping the nematode infect successfully by affecting the reproduction of *B. xylophilus.*

## 4. Materials and Methods

### 4.1. Biological Material

The highly virulent *B. xylophilus* AMA3 strain used in this study was from Anhui province, China [37]. To provide enough nematodes for the experiment, the strain AMA3 was transferred to a mycelial mat of *B. cinerea* growing on PDA plates and was cultured subsequently at 25 °C. Seven days later, the *B. xylophilus* were extracted using the Baermann funnel technique.

Two-year-old *P. thunbergii* seedlings obtained from the experimental field of Nanjing Forestry University (Jurong yaolingkou forest farm, Jiangsu, China) were used for the inoculation of the AMA3 strain. *Pinus thunbergii* seedlings were cultivated at temperatures ranging from 28 to 32 °C with relative humidity ranging from 65% to 75%. *Nicotiana benthamiana* were grown in a glasshouse at 25 °C with a relative humidity of 60% under 16:8-h light: dark conditions.

### 4.2. RNA Isolation and cDNA Synthesis

Total RNA from the nematodes was extracted using TRIzol reagent (Invitrogen) to detect the relative expression of *BxSCD3* in *B. xylophilus* at 2.5 h after inoculation. Stems of *P. thunbergii* were sampled and frozen in liquid nitrogen after inoculation with *BxSCD3* siRNA-treated and *GFP* siRNA-treated nematodes to extract RNA of *P. thunbergii*. Total RNA of *P. thunbergii* was extracted using the Plant Total RNA Kit (Zoman, Beijing, China). First-strand cDNA for RT-qPCR was synthesized from 1 μg of total RNA using HiScript II Q RT SuperMix for qPCR (+gDNA wiper) (Vazyme, Nanjing, China) according to the manufacturer’s protocol.

### 4.3. Plasmid Constructs

To determine whether BxSCD3 was an effector of *B. xylophilus*, plasmids with BxSCD3 first need to be constructed before the agrobacterium-mediated transient expression was performed. Based on *B. xylophilus* transcriptome data [21], the coding sequence of the BxSCD3 and BxSCD3sp (with a signal peptide) (BXY_0601800) were amplified from *B. xylophilus* cDNA. The BxSCD3 mutants (with nuclear localization signal (BxSCD3-NLS), a nuclear export signal (BxSCD3-NES), mutated NLS (BxSCD3-nls) and NES (BxSCD3-nes)) were amplified using combinations of primers. The amplified fragments were prepared and ligated into PVX and pBINRFP (pCAM1300-RFP), using the appropriate restriction enzymes and the Clone Express II One Step Cloning Kit (Vazyme, Nanjing), respectively. Individual colonies from each construct were tested by PCR for insertions, and the selected clones were verified by sequencing. Primer sequences are provided in Table S1.

### 4.4. Sequence Analysis

Similar sequences to BxSCD3 were retrieved by querying the BxSCD3 protein against the National Center for Biotechnology Information (NCBI) protein database using BLASTP (https://blast.ncbi.nlm.nih.gov/Blast.cgi (accessed on 5 July 2020)). The signal peptide and transmembrane helices of BxSCD3 were predicted to determine whether BxSCD3 was a candidate effector of *B. xylophilus* using the SignalP v. 5.0 server (http://www.cbs.dtu.dk/services/SignalP/ (accessed on 5 July 2020)) and the TMHMM v. 2.0 server (www.cbs.dtu.dk/services/TMHMM/ (accessed on 5 July 2020), respectively [38,39]. Domains of BxSCD3 were analyzed using SMART (http://smart.embl-heidelberg.de/ (accessed on 5 July 2020) to explore the possible known domains.

### 4.5. Agrobacterium Tumefaciens Infiltration Assays

The agrobacterium-mediated transient expression could screen and identify candidate effectors efficiently. After plasmids with BxSCD3 were constructed successfully, the transient expression was performed. The method was performed according to a previous report [7]. Briefly, the constructs were inserted into *A. tumefaciens* GV3101 by electroporation. In the agroinfiltration assays, recombinant *A. tumefaciens* strains were grown at 30 °C in a shaking incubator, at a rotation rate of 200 rpm for 12 h. Then, bacterial cells were collected by centrifugation and, subsequently, resuspended in washing buffer. The resuspended *A. tumefaciens* cells were diluted to OD600 = 0.5 for each construct. The infiltration assay was performed three times and, in each assay, three different plants with three inoculated leaves were used.

### 4.6. Subcellular Localization in N. benthamiana

To determine the subcellular localization of BxSCD3, the *N. benthamiana* leaves were agroinfiltrated with pBINRFP-BxSCD3-NLS, pBINRFP-BxSCD3-nls, pBINRFP-BxSCD3-NES, pBINRFP-BxSCD3-nes, and the P19 silencing suppressor in a 1:1 ratio at a final optical density (OD) 600 = 0.5 for each construct, respectively. Two days after agroinfiltration, patches of *N. benthamiana* leaves were cut and mounted in water, and analyzed using an LSM710 laser scanning microscope (Carl Zeiss AG, Oberkochen, Germany). RFP fluorescence was observed at an excitation wavelength of 587 nm.

### 4.7. Western Blotting

To validate the expression of tested proteins in *N. benthamiana*, the Western blotting analysis was conducted. Firstly, leaves of 4- to 6-week-old *N. benthamiana* plants were agroinfiltrated with PVX or pBINRFP genes at a final OD600 of 0.5 for each construct. Secondly, 36 h after agroinfiltration, the leaves were frozen in liquid nitrogen and ground to a fine powder using a mortar and pestle. Thirdly, total protein extraction and immunoblotting were performed referring to a previous report [40]. Transient protein expression in *N. benthamiana* was assessed by incubating the membrane with a 1:5000 dilution of a primary mouse anti-HA antibody (Abmart) or anti-RFP antibody (Abcam), followed by incubation with a goat anti-mouse secondary antibody at a 1:10,000 dilution (IRDye 800, 926-32210; LI-CORBiosciences). Finally, the proteins were visualized using an Odyssey LI-COR imaging system. Equal protein loading was confirmed by Ponceau S staining.

### 4.8. Real-Time Quantitative PCR

To detect the relative expression of *BxSCD3* at the early infection stage, the RT-qPCR was used. About 10,000 *B. xylophilus* AMA3 were inoculated into 2-year-old *P. thunbergii* seedlings; then, the nematodes were collected at 2.5 h after inoculation using the Baermann funnel technique. RNA of the *B. xylophilus* was extracted and reversely transcribed into cDNA. RT-qPCR assays were carried out using ChamQ SYBR qPCR MasterMix (Low ROX Premixed) (Vazyme) according to the manufacturer’s instructions. The actin gene of *B. xylophilus* (GenBank EU100952) was used as constitutively expressed endogenous control genes [41]. All assays were performed three times. Primer sequences are provided in Table S1.

### 4.9. In Vitro RNAi of the BxSCD3 and Inoculation Assay

To explore the function of *BxSCD3* in *B. xylophilus*, in vitro RNAi silencing of the *BxSCD3* was conducted. The siRNA soaking method was performed to silence *BxSCD3* and *GFP* according to the previous study [42]. The small interfering RNAs (siRNAs) corresponding to *BxSCD3* and the negative control GFP were synthesized, using the in vitro Transcription T7 Kit (for siRNA Synthesis) (TaKaRa), according to the manufacturer’s instructions. The nematodes were soaked in 1000 ng/µL *BxSCD3* siRNA and *GFP* siRNA solutions, and then were incubated at 20 °C in a shaking incubator with a rotation rate of 180 rpm for 48 h. The nematodes from each treatment were thoroughly washed with ddH_2_O three times, after soaking. Subsequently, approximately 2000 nematodes from two different treatments were collected to evaluate the silencing efficiency of *BxSCD3* by RT-qPCR.

In the infection assay, each 2-year-old *P. thunbergii* seedling was inoculated with approximately 1500 nematodes (a mixture of juveniles and adults) previously soaked in*BxSCD3* siRNA and *GFP* siRNA solutions, respectively. The seedlings inoculated with *GFP* siRNA-treated nematodes were used as the negative control. Based on the color of the needles, the morbidity degree of the *P. thunbergii* seedlings was classified into five different grades [43]: 0, all needles are green; I, a quarter of the needles have turned yellow; II, approximately half of the needles have turned yellow or brown; III, three-fourths of the needles have turned brown; and IV, the entire seedling has withered. The formulas [44] for calculating the infection ratio and disease severity index (DSI) of pine seedlings are indicated below:

$$\mathrm{Infection}\;\mathrm{rate}\;(\%)=\frac{\mathrm{Total}\;\mathrm{number}\;\mathrm{of}\;\mathrm{infected}\;\mathrm{plants}}{\mathrm{Total}\;\mathrm{number}\;\mathrm{of}\;\mathrm{plants}}\;\times \;100$$

$$\mathrm{Disease}\;\mathrm{severity}\;\mathrm{index}\;(\mathrm{DSI})=\frac{\sum \mathrm{the}\;\mathrm{number}\;\mathrm{of}\;\mathrm{infected}\;\mathrm{plants}\;\times \mathrm{symptom}\;\mathrm{degree}}{\mathrm{Total}\;\mathrm{number}\;\mathrm{of}\;\mathrm{plants}\;\times \mathrm{the}\;\mathrm{highest}\;\mathrm{symptom}\;\mathrm{degree}}\;\times \;100$$

The infection assay was performed three times and a total of 18 individual *P. thunbergii* seedlings for each treatment were used.

To analyze whether *BxSCD3* plays a role in the reproduction of *B. xylophilus*, each PDA plate with *B. cinerea* was inoculated with 100 individuals of the *B. xylophilus* (mixed-stage nematodes) after treatment with *BxSCD3* siRNA and *GFP* siRNA, respectively. Then, these PDA plates were cultured in the dark at 25 °C for 5 days. At the same time, each pine seedling was inoculated with 1500 individuals of the *B. xylophilus* (mixed-stage nematodes) after treatment with *BxSCD3* siRNA and *GFP* siRNA, respectively, and these seedlings were grown in the phytotron until the control plants had withered entirely. Treatment with *GFP* siRNA was used as a control. Subsequently, the Baermann funnel method was used to collect all nematodes from PDA plates and seedlings, respectively. The number of nematodes was counted with an optical microscope (Leica DM500). The two experiments above were both performed three times and each treatment had three replicates.

The total RNA of *P. thunbergii* was extracted from each segment of stems inoculated with *B. xylophilus* (mixed-stage nematodes) after treatment with *BxSCD3* siRNA for 4 h. The expression levels of three PR genes (*PtPR-3*, and *PtPR-6*) of *P. thunbergii* were detected using RT-qPCR. The elongation factor-1 alpha was used as an endogenous control [41]. This inoculation assay was performed three times, and in each assay, three different seedlings for each treatment were used.

## 5. Conclusions

In conclusion, our team identified another effector, BxSCD3, that inhibited plant immunity. This study provides information on the functional characteristics of BxSCD3, which is helpful to further understand the molecular mechanism of *B. xylophilus* causing PWD from the perspective of pathogen effectors.

## Data Availability

The data presented in this study are available on request from the corresponding author.

## Supplementary Materials

The following supporting information can be downloaded at: https://www.mdpi.com/article/10.3390/ijms23126417/s1.
