# Supplementary figures and images for: A Bursaphelenchus xylophilus Effector, BxSCD3, Suppresses Plant Defense and Contributes to Virulence
Source: Int J Mol Sci. 2022 Jun 8;23(12):6417. doi: 10.3390/ijms23126417 (PMC9223698; doi:10.3390/ijms23126417)

1dpi

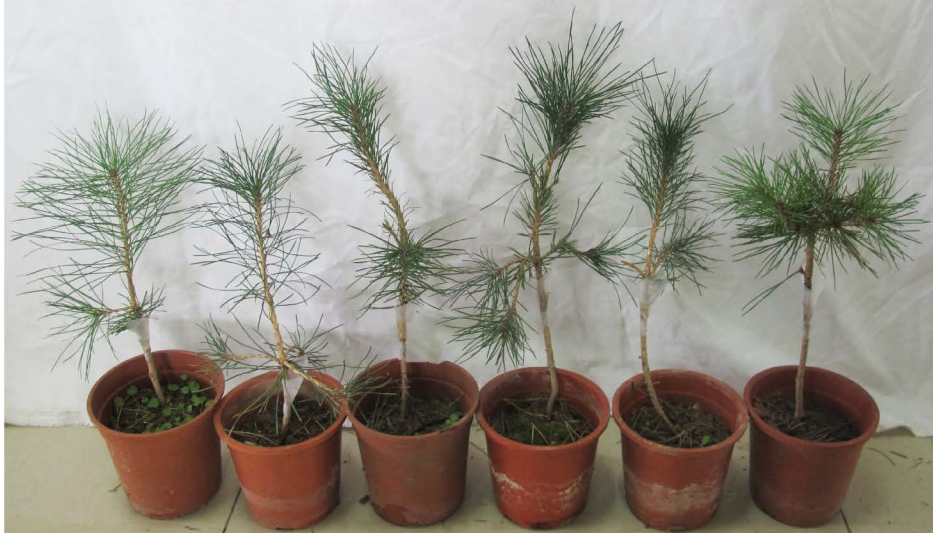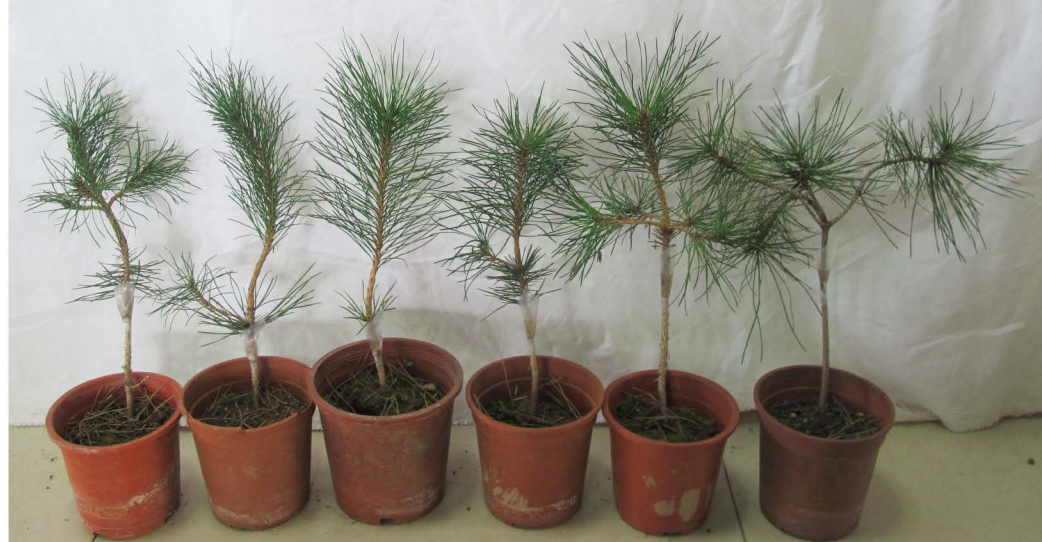

12dpi

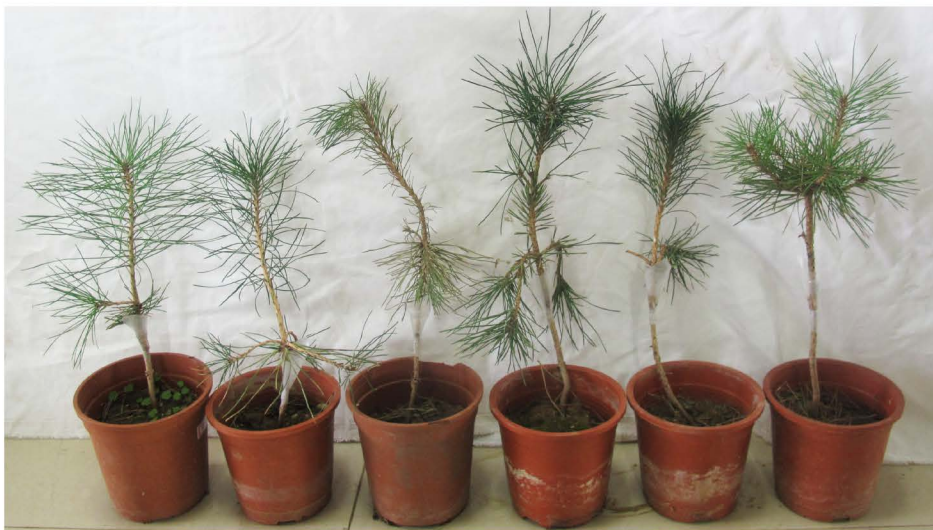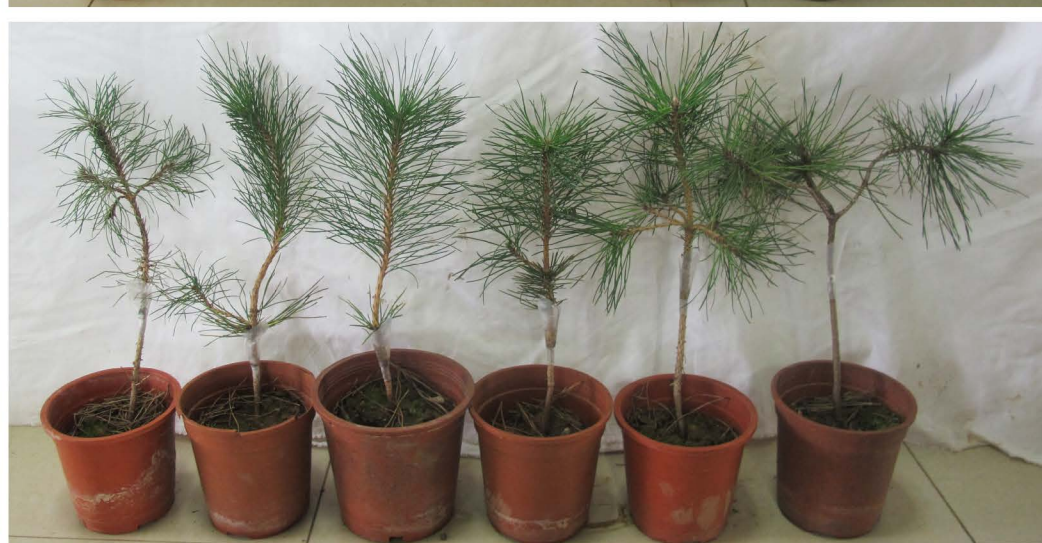

19dpi

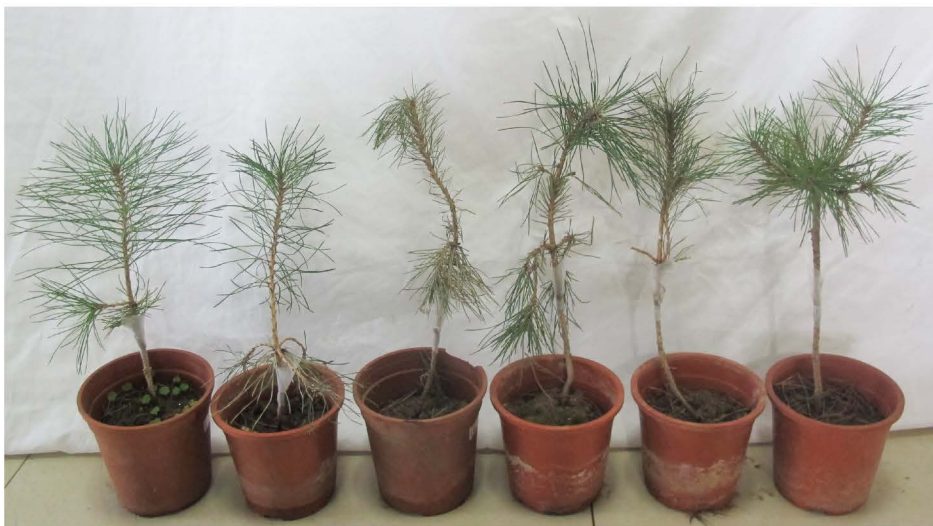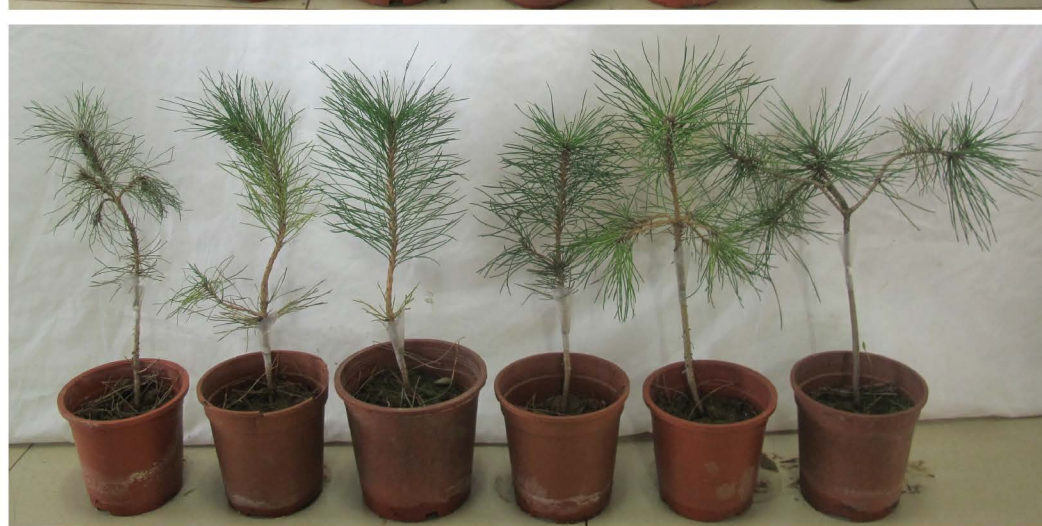

*GFP* siRNA

*BxSCD3* siRNA

Supplement: Supplementary file 1 [file ijms-23-06417-s001.zip › Figure S1.pdf]

1dpi

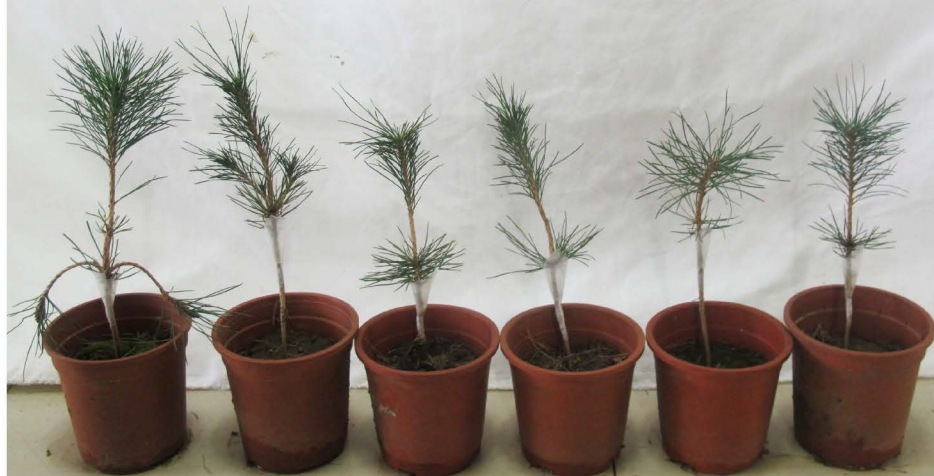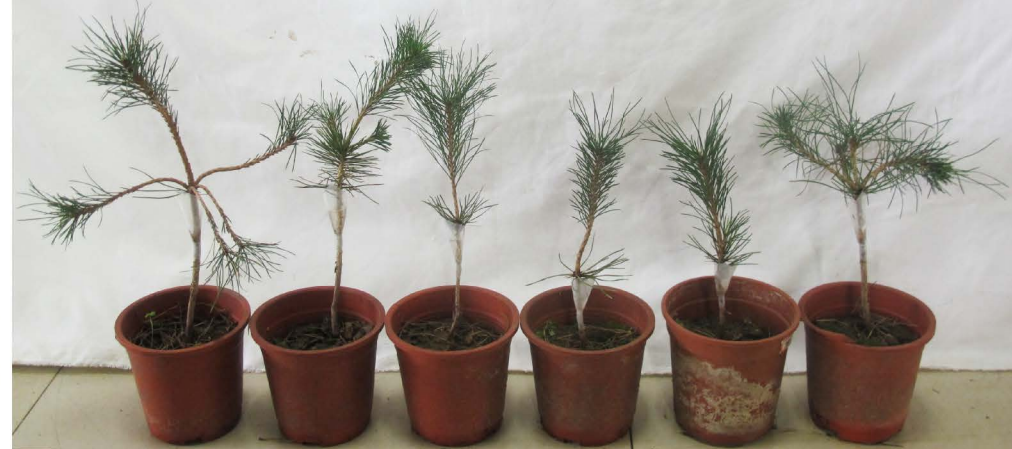

12dpi

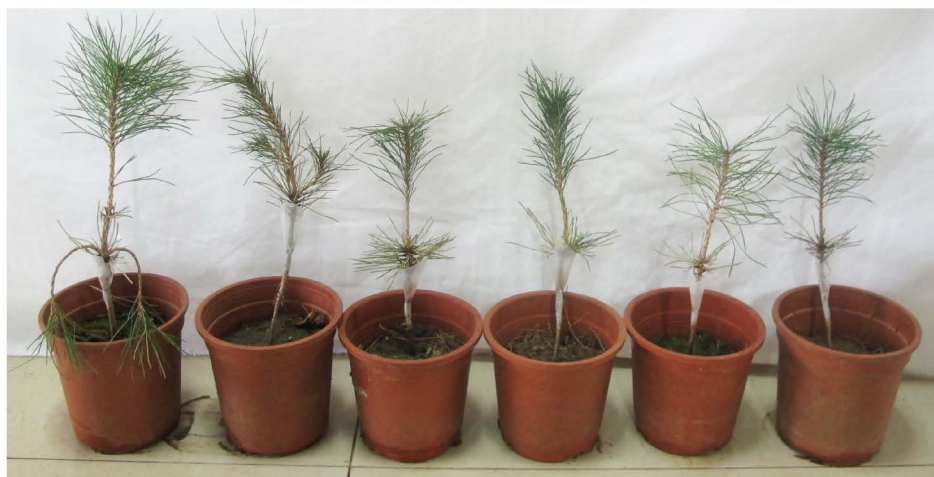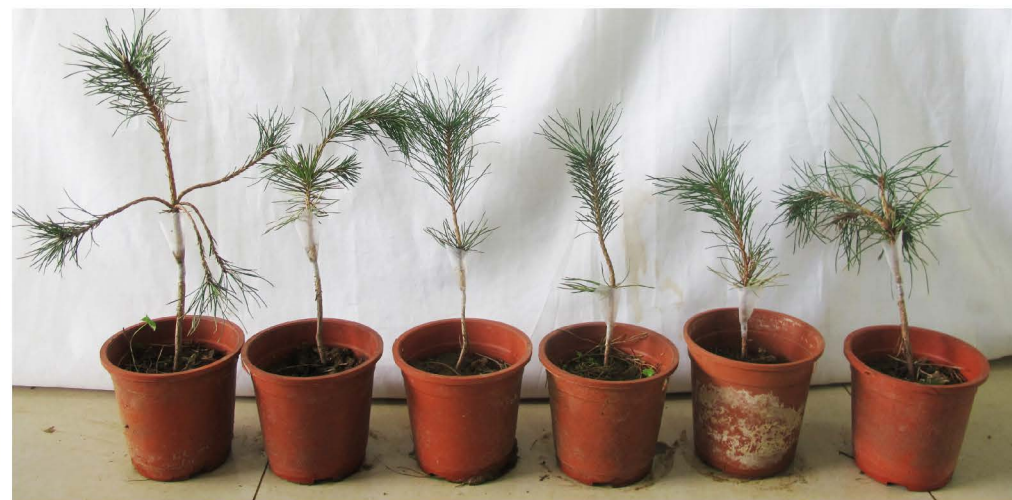

19dpi

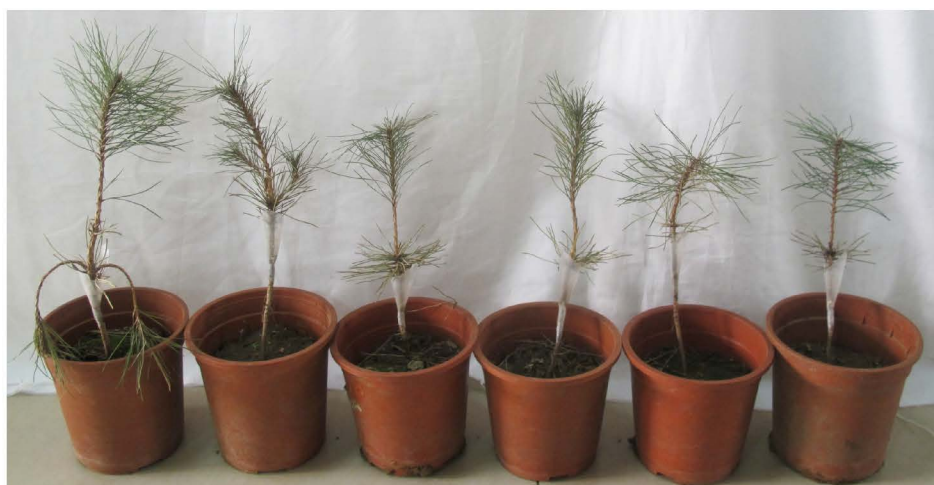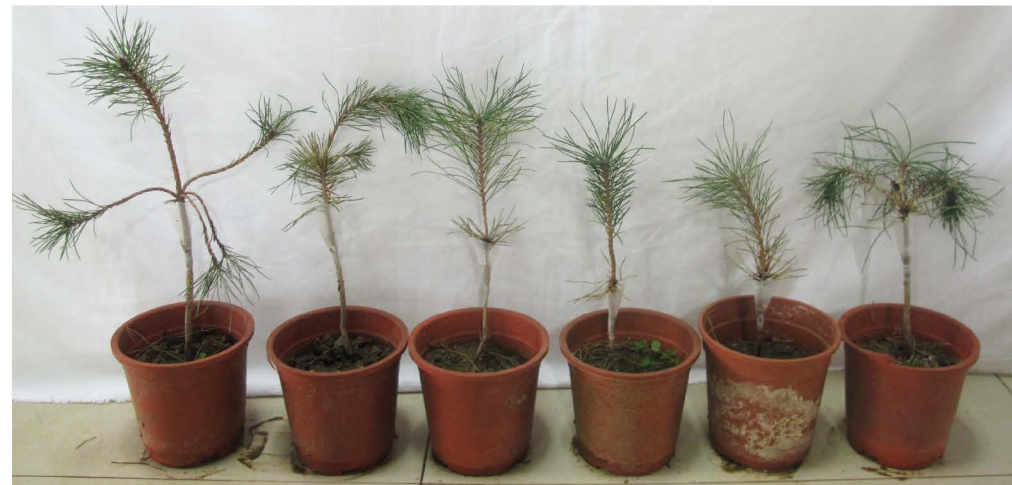

*GFP* siRNA

*BxSCD3* siRNA

Supplement: Supplementary file 1 [file ijms-23-06417-s001.zip › Figure S2.pdf]
